# Resistance Induction in Olive Tree (*Olea europaea*) Against Verticillium Wilt by Two Beneficial Microorganisms and a Copper Phosphite Fertilizer

**DOI:** 10.3389/fpls.2022.831794

**Published:** 2022-02-23

**Authors:** Ana López-Moral, Eugenio Llorens, Loredana Scalschi, Pilar García-Agustín, Antonio Trapero, Carlos Agustí-Brisach

**Affiliations:** ^1^Department of Agronomy (DAUCO, Unit of Excellence María de Maeztu 2020-23), ETSIAM, University of Córdoba (UCO), Córdoba, Spain; ^2^Departamento de Ciencias Agrarias y del Medio Natural, Universitat Jaume I de Castellón (UJI), Castellón de la Plana, Spain

**Keywords:** biological control agents, biostimulation, induced resistance, priming, *Verticillium dahliae*

## Abstract

Enhancement of the natural defenses of a plant by beneficial microorganisms, i.e., endophytic bacteria and fungi or fertilizers such as copper phosphonates, could result in a potential alternative strategy against verticillium wilt of olive tree (*Olea europaea*). In this study, two beneficial microorganisms (the fungus *Aureobasidium pullulans* AP08 and the bacterium *Bacillus amyloliquefaciens* PAB-024) and a phosphonate salt copper phosphite (CuPh) were evaluated for their effectiveness as host resistance inducers against *Verticillium dahliae* in olive. To this end, 6-month-old healthy olive plants of the susceptible cultivar Picual were treated by foliar or root applications by spraying 15 ml per plant or by irrigation with 350 ml per plant of the dilutions of each product (CuPh: 3 or 10 ml l^–1^, respectively; PAB-024: 10^8^ UFC ml^–1^; AP08: 10^6^ UFC ml^–1^). Treatments were conducted weekly from 2 weeks before inoculation to 10 days after inoculation. A cornmeal–water–sand mixture (1:2:9; w:v:w) colonized by *V. dahliae* was used for plant inoculation. Additionally, treated and noninoculated, nontreated and inoculated, and nontreated and noninoculated plants were included for comparative purposes. Disease severity progress and shoot fresh weight were assessed. Parameters involved in plant resistance were monitored through determination and quantification of reactive oxygen species (ROS) response (H_2_O_2_), and evaluation of hormones was done by gene expression analysis. *Aureobasidium pullulans* and CuPh were the most effective in disease reduction *in planta* by foliar or root application, respectively. Plants treated with CuPh showed significantly higher shoot fresh weight compared to the other treatments. ROS was significantly enhanced in plants treated with *B. amyloliquefaciens* PAB-024 compared to the rest of treatments and control. With regard to the evaluation of hormones, high levels of salicylic acid were detected on leaves from all treatment combinations, but without significant enhancements compared to the nontreated control. Regarding the gene expression related to salicylic acid, only the *WRKY5* gene has shown a strong enhancement in the treatment with *B. amyloliquefaciens*. On the other hand, a strong accumulation of jasmonic acid and jasmonic acid-isoleucine in plants treated with *A. pullulans* was observed in all the tissues analyzed and also in the roots of plants treated with *B. amyloliquefaciens* and CuPh.

## Introduction

Verticillium wilt of olive (*Olea europaea* subsp. *europaea* L.) (VWO), caused by the hemibiotrophic soil-borne fungus *Verticillium dahliae* Kleb., is considered the most important disease of this crop worldwide due to the high levels of tree mortality and fruit yield reduction that it causes (López-Escudero and Mercado-Blanco, 2011; Jiménez-Díaz et al., 2012; Montes-Osuna and Mercado-Blanco, 2020). This fact is not only because of the difficulty to control this pathogen, which can survive for up to 14 years in the soil, forming microsclerotia (MS) (Pegg and Brady, 2002) or as endophyte in the xylem vessels of the infected plants (Alström, 2001), but also because of the lack of effective chemical treatments against the disease (Trapero et al., 2015; Mulero-Aparicio et al., 2020). Thus, based on the advances about the control of VWO conducted over the last two decades, there is no doubt that an integrated management strategy is needed to reduce both pathogen dispersal and disease incidence in olive orchards (López-Escudero and Trapero, 2017; Montes-Osuna and Mercado-Blanco, 2020). Within this strategy, the combination of several ecofriendly control methods such as genetic resistance, cultural practices, and biological control in both pre- and postplanting must be considered (López-Escudero and Mercado-Blanco, 2011; Trapero et al., 2015; Montes-Osuna and Mercado-Blanco, 2020; Ostos et al., 2020; López-Moral et al., 2021).

With respect to biological control, a wide diversity of compounds including organic amendments, essential oils, seaweeds, plant or microorganism extracts, plant biostimulants, fertilizers, mycorrhizal fungi, and antagonist microorganisms, i.e., endophytic bacteria and fungi, have been evaluated worldwide over the last two decades as a potential ecofriendly control measure against VWO (Montes-Osuna and Mercado-Blanco, 2020; López-Moral et al., 2021; Poveda and Baptista, 2021). Most of these studies have been conducted under controlled experimental conditions both *in vitro* and *in planta*. Nevertheless, the efficacy of most of these compounds has not yet been demonstrated against VWO under natural conditions in olive groves. To our knowledge, only a few studies reveal high or moderate effectiveness of some of these compounds against VWO in the field, e.g., the bacterium *Paenibacillus alvei* strain K165 (Markakis et al., 2016), the nonpathogenic fungus *Fusarium oxysporum* strain FO12 and a commercial mix of *Trichoderma asperellum* and *T. gamsii* (Mulero-Aparicio et al., 2020), *T. harzianum* (Arici and Demirtas, 2019), and several mycorrhizal fungi including diverse *Glomus* spp. (Porras-Soriano et al., 2006) and *Rhizophagus irregularis* (Arici and Demirtas, 2019). In addition, the results obtained from studies on the biocontrol of VWO suggest that all these evaluated compounds could be acting against the pathogen by means of several modes of action including soil suppression, endophytism, antagonism, and host resistance induction (HRI) (Tjamos et al., 2005, 2004; Ruano-Rosa et al., 2017; Varo et al., 2018; Mulero-Aparicio et al., 2019a,b; Azabou et al., 2020; Montes-Osuna and Mercado-Blanco, 2020; López-Moral et al., 2021; Poveda and Baptista, 2021).

Regarding the efficacy of the phosphites in crop protection, experimental evidence from decades has shown its positive effects on plant metabolism, which are more evident when applied to the roots in hydroponic systems or to the leaves by spraying. Their efficacy has been demonstrated not only as a fertilizer by increasing plant growth, production, and productivity, but also as a pesticide against various species of pathogenic bacteria, fungi, and oomycetes (Guest and Grant, 1991; Cooke and Little, 2002; Abbasi and Lazarovits, 2006a,b; Gómez-Merino and Trejo-Téllez, 2015; Romero et al., 2019). Moreover, phosphites have also emerged as a potential HRI, as it has demonstrated their effectiveness against different biotic and abiotic stress factors improving crop protection, yield, and quality (Navarro-Cerrillo et al., 2004; Gómez-Merino and Trejo-Téllez, 2015; Barros-Rodríguez et al., 2020). Among the diversity of modes of action that all these compounds may perform preventing plant pathogen infection, their capability to induce the natural plant defenses by triggering a systemic resistance through jasmonic acid (JA)/ethylene (ET)- and/or salicylic acid (SA)-dependent signaling pathways has been currently considered as one of the most promising ecofriendly strategies against plant diseases (Llorens et al., 2017a,b; Poveda and Baptista, 2021). In this way, HRI stimulates the defensive plant response by means of appropriate natural or chemical compounds and plants become tolerant against both biotic and abiotic stresses (Conrath, 2009; Llorens et al., 2017b; Poveda and Baptista, 2021). The stimuli not only induce the innate resistance of the plant for overcoming the attack of the pathogen, but can also persist in the plant with long-term effects preventing new infections (Bektas and Eulgem, 2015; Llorens et al., 2017b). For these reasons, besides the low or null toxicity that the host resistance inducers have for the environment (González-Hernández et al., 2018), HRI deserves to be explored as a potential biocontrol tool against VWO. Indeed, the effect of several compounds such as SA (Gharbi et al., 2016) or seaweed extracts (Salah et al., 2018) which are able to enhance the olive plant resistance against *V. dahliae* has been already demonstrated *in planta* with promising results. Moreover, the ability of the endophytic bacteria and fungi acting as HRIs against plant infections by the major olive pathogens has been already demonstrated in several plant models such as *Arabidopsis thaliana*, *Brassica napus* or *Solanum lycopersicum* (Poveda et al., 2019; Montes-Osuna and Mercado-Blanco, 2020; Poveda and Baptista, 2021). In the particular case of VWO, Tjamos et al. (2005) demonstrated that the bacterium *Paenibacillus alvei* K165 mediates the HRI genes related to the components of the isochorismate pathway and a functional nonexpressing NPR1 protein since they were expressed in *Arabidopsis* mutants and transgenic plants impaired in defense signaling pathways. In addition, Schilirò et al. (2012) showed that many genes related to plant defense and response to different stresses were expressed by suppression subtractive hybridization in olive plants after root colonization by *Pseudomonas fluorescens* PICF7. In this same study, the induction of lipoxygenase, phenylpropanoid, terpenoids, and plant hormones biosynthesis transcripts in PICF7-colonized olive roots was confirmed by quantitative real-time PCR (qRT-PCR) analyses (Schilirò et al., 2012). Nevertheless, a much in-depth knowledge of the mechanisms related to HRI to VWO based on molecular and physiological analyses is needed to better determine the effectiveness of natural compounds as resistance inducers of olive plants against *V. dahliae*.

In spite of the literature cited above, the regulation of SA-, JA-, or ET-related genes enhancing innate olive resistance to *V. dahliae* through treatments by beneficial microorganisms and plant biostimulants is still poorly understood. Within this context, previous studies evaluating 32 potential HRIs against VWO showed that two beneficial microorganisms (the fungus *Aureobasidium pullulans* AP08; and the bacterium *Bacillus amyloliquefaciens* PAB-24) and a phosphonate salt copper phosphite (CuPh) were highly effective in reducing the disease progress *in planta* (López-Moral et al., 2021). It is worth mentioning that *A. pullulans* AP08 did not show a direct effect on the pathogen since it did not inhibit mycelial growth and MS germination of *V. dahliae*, while it resulted in the most effective treatment in reducing the disease progress (López-Moral et al., 2021). The latter result suggested that *A. pullulans* AP08 could act against the pathogen through different modes of action such as antagonism or HRI. However, the methodology used in this previous study was not enough to determine their mode of action. Therefore, the main goal of this study was to elucidate the effectiveness of *A. pullulans* AP08, *B. amyloliquefaciens* PAB-024, and CuPh in enhancing the plant defense system in olive plants (‘Picual’) against *V. dahliae*. To this end, inoculated or noninoculated, and treated and nontreated plants combinations per each compound were monitored for disease severity and plant growth. In parallel, biochemical parameters involved in plant resistance, i.e., reactive oxygen species (ROS) response (H_2_O_2_) and plant hormones were validated by gene expression qRT-PCR analysis.

## Materials and Methods

### Plant Material and Growth Conditions

Healthy 6-month-old olive potted plants of cv. Picual (highly susceptible to *V. dahliae*; López-Escudero et al., 2004) growing in peat moss into plastic pots (0.5 l) from a commercial nursery were used in this study. Prior to start the experiments, plants were maintained in a controlled-growth chamber at 22 ± 2°C in a 14:10 h (light:dark) photoperiod of white fluorescent light (10,000 lux) and 60% relative humidity (RH) for 1 month, and irrigated three times per week with 300 ml of water per plant.

### Plant Treatments, Inoculation, and Experimental Design

After 1 month under the preconditioning conditions described above, plants were treated with the BCAs *Aureobasidium pullulans* AP08, *Bacillus amyloliquefaciens* (PAB-024), and with the commercial phosphonate salt copper phosphite (CuPh; Phoscuprico^®^, AGRI nova Science, Almería, Spain). The two microorganisms belong to the collection of the Department of Agronomy of the University of Córdoba (Spain). The fungus *A. pullulans* AP08 is maintained as single-spore culture on potato dextrose agar (PDA; Difco Laboratories^®^, MD, United States) slants with sterile paraffin oil (Panreac Química SA, Barcelona, Spain) at 4°C in the dark; and the bacterium *B. amyloliquefaciens* PAB-024 is cryopreserved with 30% glycerol at –80°C. An aqueous inoculum was prepared for the two microorganisms. For AP08, fresh colonies were obtained from the stock growing on PDA at 23 ± 2°C for 7 days in a 12:12 h (light:dark) photoperiod. Then, the surface of the colonized Petri dishes were covered with sterile distilled water, scraped from the medium with a sterile glass rod, and filtered through a sterile gauze. For PAB-024, 20-μl of the stock was introduced in a 250-ml Erlenmeyer flask with 100 ml of nutrient broth (NB; Difco Laboratories^®^, MD, United States), and incubated at 23 ± 2°C for 2 days in a 12:12 h (light:dark) photoperiod in an orbital shaker. Subsequently, 1 ml of the microorganisms solutions was introduced in a 2-l Erlenmeyer flask containing 1 l of sterile potato dextrose broth (PDB; Difco Laboratories^®^, MD, United States) or 1 l of sterile NB for AP08 and PAB-024, respectively, and incubated at 23 ± 2°C for 3 days in a 12:12 h (light:dark) photoperiod in an orbital shaker. Finally, *A. pullulans* AP08 and *B. amyloliquefaciens* PAB-024 suspensions were adjusted at 10^6^ conidia ml^–1^ and 10^8^ CFU ml^–1^, respectively, being used as the final inoculum solutions to conduct the plant treatments. Regarding the commercial product, CuPh was applied at the maximum irrigation doses recommended by the manufacturer (3- and 10 ml l of water^–1^ for foliar and root applications, respectively; see below) (López-Moral et al., 2021).

Plants were treated by spraying 15 ml (foliar application) or by irrigation, adding 300 ml (root application) per plant of the respective solution or suspension of each compound described above or water. Treatments were conducted as follows: (i) foliar applications, 14, 13, and 11 weeks before plant sampling (see below); (ii) irrigation applications, 13 and 11 weeks before plant sampling (see below). There were two independent plant lots per treatment and type of application (foliar or root) combination: (i) noninoculated plants; and (ii) plants that were inoculated with *V. dahliae* 8 weeks before sampling. Additionally, lots of nontreated and noninoculated, and nontreated and inoculated plants were also included as negative and positive controls, respectively.

The *V. dahliae* inoculum was prepared using 2-l Erlenmeyer flasks filled with 1 kg of a cornmeal–sand mixture (750 g of sand, 83 g of cornmeal, and 167 ml of distilled water). Then, they were double-sterilized on two consecutive days at 120°C for 50 min (1st day) and 120°C for 20 min (2nd day), with the flasks being manually shaken between each sterilization step. Subsequently, 50 mycelial plugs (7.5 mm in diameter) of *V. dahliae* isolate V180 growing on PDA at 23 ± 2°C for 14 days in the dark were introduced into each flask, and the flasks were incubated at 24°C in darkness for 4 weeks. To favor the homogeneous colonization of the cornmeal–sand mixture by the pathogen, flasks were manually shaken just after inoculation as well as once a week during the incubation period. After 4 weeks of incubation, the inoculum density of the colonized cornmeal sand was estimated by means of the serial dilution method on PDA, and expressed as colony-forming units (CFUs). Then, a mix of the colonized cornmeal–sand mixture *V. dahliae* and sterile peat most was adjusted to 10^7^ CFU g^–1^ of the final substrate (López-Moral et al., 2021), which was used to conduct plant inoculation.

For inoculation, plants were taken out of the plastic pots, the original peat moss was carefully removed from the roots, and plants were transplanted in 0.7 l plastic pots filled with the adjusted final substrate described above. Just after inoculation, plants were immediately irrigated with distilled water and incubated in a growth chamber at 20 ± 2°C at 100 RH in the dark for 4 days. Subsequently, light and humidity parameters were progressively modified over 1 week until reaching 23°C, a 12-h photoperiod of fluorescent light (10,000 lux), and 70% RH, which were maintained until the end of the experiment. Moreover, after the last root treatment application, plants were irrigated three times per week with 300 ml of water per plant until the end of the experiment.

A factorial randomized complete block design (three blocks) was used with treatments (*n* = 4; Water treated control, AP08, PAB-024 or CuPh), type of application (*n* = 2; spray or irrigation), and inoculation with *V. dahliae* (*n* = 2; inoculated or noninoculated plants) as independent variables and five replicated olive plants per treatment and block (4 × 2 × 2 × 3 × 5 = 240 plants in total). The experiment was maintained for 12 weeks after inoculation.

### Disease Severity and Plant Growth Evaluation

Disease severity (DS) was periodically assessed from symptoms onset to 12 weeks after inoculation when the disease progress stopped. A 0–4 rating scale of 17 values was used to estimate the percentage of affected tissue of the aerial part of the plants by means of five main categories of severity: 0 = no symptoms; 1 = 25%, 2 = 50%, 3 = 75%, and 4 = 100% (dead plant) of affected tissue, with three intermediate values (0.25, 0.50, and 0.75) between main categories (Varo et al., 2018). Therefore, the scale values (*X*) were related to the percentage of affected plant (*Y*) according to the equation: *Y* = 25*X*. DS data were used to calculate the relative area under the disease progress curve (RAUDPC) at the end of the experiment by the trapezoidal integration method (Campbell and Madden, 1990). Additionally, disease incidence (DI) and mortality were also estimated as the percentage of symptomatic or dead plants, respectively, at the end of the experiment.

In parallel, the fresh weight of the leaves and stems of each plant was measured at the end of the experiment to estimate the effect of biological treatment on the plant growth development. For each type of tissue (leaf or stem), data of the five plants of each block were averaged.

### Assessment of the Biochemical Parameters Involved in Plant Resistance

#### Sampling Plant Material

For both foliar and root lots of treated plants of each compound tested, one replicated plant per block and treatment combination (treated and noninoculated; treated and inoculated) was randomly selected at the end of the experiment (3 months after inoculation). One random plant per block of the respective controls (nontreated and inoculated; nontreated and inoculated) was also sampled. A total of three plants per treatment or control combination were used. In each plant, leaves were carefully separated from the shoots and stem, and sampled as separated tissues (leaves and stems) for further analysis. Roots of the same plants were also sampled. The plant material was subjected to different protocols as described below depending on the biochemical parameters evaluated in this study.

#### Determination and Quantification of Hydrogen Peroxide

For each compound, and treatment and control combinations, samples of 10 leaves and 10 stem segments (≈5 cm in length) were collected at 11 weeks after the last spray or irrigation application. Tissues collected from the two sampled plants were immersed immediately in independent recipients in 1 mg ml^–1^ of 3’,3-diaminobenzidine (DAB, Merck KGaA, Darmstadt, Germany) at pH < 3 for 24 h in darkness. Subsequently, they were discolored in 96% ethanol and rehydrated in distilled water for 30 min. To determine the ROS, the formation of brown precipitates were quantified in micrographic as described by Llorens et al. (2017a), and expressed as the number of dark-brown DAB pixels with respect to the total pixels corresponding to plant material using the GIMP program (version 2.6.12).

#### Evaluation of Hormones Related to Plant Defense

For each compound and treatment and control combinations, a third lot of samples of 10 leaves and 10 shoot-stem segments (≈5 cm in length) was collected at 11 weeks after the last spray or irrigation application. Additionally, the root system of each plant was also sampled in its integrity at the same time. All the fresh plant material was immediately frozen in liquid N, maintained at –20°C for at least 24 h in independent plastic bags, and then lyophilized at –43°C, ≈0.344 mBar (Telstar^®^ Cryodos, Azbil Group, Terrassa, Spain) for 10–14 days. Subsequently, dry tissue (0.05 g) of each sample was homogenized in 1.5 ml of ultrapure water, and 100 μL of internal standard at 100 ng ml^–1^ {deuterated SA ([_2_H_4_]), dihydrojasmonic acid (dhJA), and propylparaben} were added prior to extraction to quantify the level of hormones JA, JA-isoleucine (JAIle), 12-oxo-phyto dienoic acid [OPDA], and SA). The samples were centrifuged at 5,000 rpm for 45 min at 4°C. The supernatant was partitioned against diethylether, dried in a speed vacuum, and then resuspended in 90:10 H_2_O:MeOH. After extraction, a 20 μl aliquot of the homogenized suspension of each sample was injected directly into the ultrahigh-performance liquid chromatography (UPLC) system and analyzed by means of a Waters Alliance 2690 HPLC system (Milford, MA, United States) with a nucleosil ODS reversed-phase column (100 mm × 2 mm, i.d., 5 μm; Scharlab, Barcelona, Spain).^1^ The chromatographic system was interfaced to a Quatro LC (quadrupole--hexapole--quadrupole) mass spectrometer (Micromass).^2^ Finally, the quantitative data from calibration standards and plant samples was conducted using the MASSLYNX NT software version 4.1 (Micromass) (Llorens et al., 2017a).

We can consider that the absence of hormones for certain treatment combinations is due to the fact that they are at a concentration below the UPLC limit of quantification. Despite the hormones being present, and we can observe a signal in the chromatogram, the method of peak integration and calculation based on the peak of internal standard and curve of calibration makes it not possible to obtain realistic values below 1 ppb.

#### Data Validation and Expression Profile Analysis by Quantitative Real-Time PCR

Leaf, stem and, root tissue samples were obtained as described above, immediately frozen in liquid nitrogen and kept at –80°C until RNA extraction. Total RNA was extracted using E.Z.N.A Plant RNA kit (OMEGA biotek)^3^, according to the manufacturer’s instructions. A total of 1 μg of total RNA after DNase treatment (Promega)^4^ was reverse transcribed into cDNA using an oligodT primer and primescript RT enzyme mix 1 (Primescript RT reagent kit, TaKaRa Bio Europe SAS, Saint-Germain-en-Laye, France). Finally, the mRNA purity and quality were checked by spectrophotometry using a NanoDrop 2000 Spectrophotometer (Thermo Scientific, Waltham, MA, United States).

Quantitative real-time PCR experiments were conducted to validate the expression of hormones related to plant defense identified in the previous biochemical analysis. To determine whether plant treated by CuPh or treated and colonized by AP08 or PAB-024 strains triggers defense responses in olive tissues, several genes identified as induced and putatively involved in the synthesis of acetone cyanohydrin lyase (*ACL*) and lipoxygenase (*LOX*), as well as the transcription factors involved in systemic defensive responses *WRKY5*, i.e., SA signal transduction and basic helix—loop–helix (*bHLH*) or factors responsive to jasmonic acid, were selected for validation by qRT-PCR analysis (Schilirò et al., 2012). The specific primer pairs used to amplify each gene are shown in Table 1. The range of concentrations at which target cDNA and cycle thresholds (Ct) values were determined by linear correlation; and specific qRT-PCR assays were conducted using cDNAs synthesized from 10-fold serially diluted (1 mg, 100 ng, 10 ng, 1 ng, and 100 pg) RNA simples to estimate PCR efficiencies. For each selected gene, relative standard curves were constructed using reverse transcribed cDNA from serial dilutions of total RNA of a “Reference Sample” of the same pooled total RNA used for the subtraction experiment. Ct values and the logarithm of cDNA concentrations were linearly correlated for each of the examined genes. For all transcripts, reactions were repeated twice (independent qRT-PCR experiments), and three biological replicates per transcript and per plate were included in all cases.

**TABLE 1:** Transcripts used in this study.

Clone ID^a^	Putative Gene	Process	Primer pair^c^	Amplicon length (bp)
ARBRI-C140	Acetone cyanohydrin lyase (*ACL*)	Salicylic acid-binding protein 2	Fw: GAAAGAGATGGAAGCGGAAA; Fv: ACACAGGGAAATGCATCAAA	246
ARBRI-2_T7_F05	Lipoxygenase (*LOX*)	Jasmonic acid biosynthesis	Fw: TATCTCCCGGTTCGTCCTAC; Rv: CCTCAACTTCGTCCCAAATC	264
ARBRI-C83	*WRKY5*	SA signal transduction	Fw: GCATGGTGCAAGAAGTAGGA; Rv: CAGCAACAAACGCTACACCT	213
ARBRI-C74	*bHLH*	JA-responsive transcription	Fw: TTACAGCGCAGAATCCCTAA; Rv: TGTGCGGGGGAATATAGAAT	213
AF545569^b^	*Olea europea beta-actin* (*olive act1*)	Citoskeletal integrity	Fw: GCTTGCTTATGTTGCTCTCGAC; Rv: TGATTTCCTTGCTCATACGGTC	308

*^a^All transcripts were selected and analyzed according to Schilirò et al. (2012).*

*^b^Olive act-1 gene used as reference to normalize relative expression (Gene Bank Accession Number).*

*^c^Fw, forward; Rv, reverse.*

Quantitative real-time PCR was performed with Maxima SYBR Green/ROX qPCR Master Mix (Thermo Fisher, MA, United States) in a StepOne™ Real-Time PCR System (Thermo Fisher, MA, United States). The following thermal cycling profile was used for all PCR reactions: 94°C for 5 min, 50 cycles of 94°C for 30 s, 55°C for 30 s, and 72°C for 40 s. Linear equations, determination coefficients (*R*^2^), and reaction efficiencies were calculated in all cases. Melting curves of qRT-PCR products were assessed from 55 to 95°C to confirm the amplification of single PCR products. The *Olea europaea b-actin* gene (*olive act1*) was used as a housekeeping gene and amplified under the same conditions in order to normalize the data resulting from qRT-PCR and to calculate the relative expression (RE) levels (Livak and Schmittgen, 2001; Schilirò et al., 2012).

### Data Analyses

The experiment was conducted twice, and data from the two repetitions of the experiment were combined after checking for homogeneity of the experimental error variances by the *F* test (*P* ≥ 0.05). Then, data were tested for normality, homogeneity of variances, and residual patterns, and they were logarithmically transformed when necessary. To evaluate the effect of treatments on disease progression and plant growth in inoculated plants, factorial ANOVAs were conducted with RAUDPC (%) or DS (%) as dependent variables, and treatments [*n* = 5; 3 products + 2 control (positive and negative)], type of application (*n* = 2; foliar and irrigation) and their interaction as independent variables. Since interaction was significant in both cases (*P* < 0.05), two separate one-way ANOVAs were conducted for each type of application (foliar or irrigation). Data on the final DI (% of affected plants) and mortality (% of dead plants) were analyzed by multiple comparisons for proportions tests at *P* = 0.05 (Zar, 2010). Regarding the fresh weight (g) data from the leaf and stem tissues, two separate factorial ANOVAs, which included inoculated or noninoculated plants and treatments, for each type of application were conducted since the interactions between the type of application and treatments were significant (*P* < 0.05). To evaluate the effect of compounds on the expression of parameters related to HRI (ROS, hormone levels, and gene expression), a factorial ANOVA was conducted for each olive tissue (leaf, stem, or root) combination, with ROS, SA, JA, JA-Ile, OPDA, *LOX*, *ACL*, *WRKY5*, or *bHLH* as dependant variables and the compound (AP08, PAB-024, or CuPh), the type of application (foliar or root) and inoculation (inoculated or noninoculated plants), and their respective interactions as independent variables. When the interactions were significant, they were used to compare the treatment means. Mean values were compared using Fisher’s protected LSD test for experiments with independent variables with less than six levels or Tukey’s HSD tests for experiments with independent variables with six or more levels, both at *P* = 0.05 (Steel and Torrie, 1985). Data from this study were analyzed using Statistix 10.0 software (Analytical Software, 2013).

## Results

### Effect of Treatments on Verticillium Wilt Development in Olive Plants

Symptoms of verticillium wilt disease were only observed in inoculated plants, so noninoculated plants were excluded from this analysis. In inoculated plants, disease symptoms developed from 4 to 12 weeks after inoculation, when the progress of the disease was stopped. The results of the effect of the foliar or root applications on the parameters of disease are summarized in Table 2. When the treatments were applied by spraying, *A. pullulans* AP08 was the most effective treatment showing significantly lower values, both for DS (42.7 ± 2.9%) and for RAUDPC (36.4 ± 2.7%), compared to the rest of the treatments. However, *B. amyloliquefaciens* PAB-024 and CuPh showed a similar effect between them for DS and RAUDPC, but only for RAUDPC they showed a significant effect compared with the nontreated and inoculated control. Concerning the DI, plants treated with *B. amyloliquefaciens* PAB-024 showed 100% of DI, whereas the mortality (46.8%) was significantly lower in comparison with the nontreated and inoculated control (100%). *Aureobasidium pullulans* AP08 and CuPh treatments showed a similar reduction on DI (80.4 and 87.9%, respectively), but *A. pullulans* AP08 treated plants showed the lowest mortality (14.0%) (Table 2).

**TABLE 2:** Disease-related parameters for olive plants grown in artificially infested substrate with the defoliating *Verticillium dahliae* isolate V180 and treated with several products by spraying (foliar application) or irrigation (root application)^a^.

Foliar application				

Treatment	Incidence (%)^b^	Mortality (%)^b^	Disease severity (%)^c^	RAUDPC (%)^d^
*Aureobasidium pullulans*	80.4 b	14.0 c	42.7 ± 2.91 b	36.4 ± 2.7 c
*Bacillus amyloliquefaciens*	100.0 a	46.8 b	83.5 ± 6.9 a	65.9 ± 6.4 b
Copper phosphite	87.9 b	41.1 b	81.3 ± 9.8 a	64.6 ± 8.5 b
Water (inoculated)	100.0 a	100.0 a	100.0 ± 0.0 a	100.0 ± 0.0 a
Water (noninoculated)	0.0 c	0.0 d	0.0 ± 0.0 c	0.0 ± 0.0 d

**Root application**				

**Treatment**	**Incidence (%)^b^**	**Mortality (%)^b^**	**Disease severity (%)^c^**	**RAUDPC (%)^d^**

*Aureobasidium pullulans*	77.3 b	4.6 c	32.1 ± 1.6 b	25.6 ± 2.6 b
*Bacillus amyloliquefaciens*	80.0 b	17.8 b	41.5 ± 3.3 b	27.3 ± 6.6 b
Copper phosphite	51.5 c	2.8 c	22.7 ± 2.9 b	20.6 ± 5.2 b
Water (inoculated)	100.0 a	100.0 a	100.0 ± 0.0 a	100.0 ± 0.0 a
Water (noninoculated)	0.0 d	0.0 c	0.0 ± 0.0 c	0.0 ± 0.00 c

*^a^Olive plants were treated several times before and after inoculation and disease parameters were assessed weekly from 4 to 12 weeks after inoculation with V. dahliae. Water treatments in inoculated or in noninoculated plants were used as positive or negative controls, respectively.*

*^b^Percentage of symptomatic plants (Incidence) or dead plants (Mortality) 12 weeks after planting in the infested substrate with V. dahliae isolate V180 (n = 30). For each parameter and type of application, means in a column followed by the same letter do not differ significantly according to Zar’s multiple comparisons for proportions test at P = 0.05 (Zar, 2010).*

*^c^Final disease severity ± Standard Error of the means (SE) 12 weeks after planting in the infested substrate with V. dahliae isolate V180. Disease severity was assessed using a 0–4 rating scale of 17 values, in which the scale values (X) were related to the percentage of affected plant (Y) according to the equation: Y = 25X. For each type of application, means in a column followed by the same letter do not differ significantly according to Fisher’s protected LSD test at P = 0.05 (Steel and Torrie, 1985).*

*^d^Relative area under the disease progress curve (RAUDPC) ± SE developed over the assessment period. For each type of application, means in a column followed by the same letter do not differ significantly according to Fisher’s protected LSD test at P = 0.05 (Steel and Torrie, 1985).*

On the other hand, when treatments were applied by irrigation, there was a significant reduction in RAUDPC and DS for the three products compared with the nontreated and inoculated control, but no differences were observed between them. Although a similar effect was observed among the three products, CuPh showed the lowest values of RAUDPC (20.6 ± 5.2%) and DS (22.7 ± 2.9%). Regarding the DI, CuPh was also the most effective treatment (DI = 51.5%), whereas *A. pullulans* AP08 (DI = 77.3%) and *B. amyloliquefaciens* PAB-024 (DI = 80.0%) showed a lower effect between them. Despite the fact that the DI for the plants treated with two microorganisms was high, the mortality was significantly lower for all the tested products compared with the nontreated and inoculated control, with a range between 17.8 and 2.8% for *B. amyloliquefaciens* PAB-024 and CuPh, respectively (Table 2).

### Effect of Treatments on Plant Growth

**FIGURE 1:**
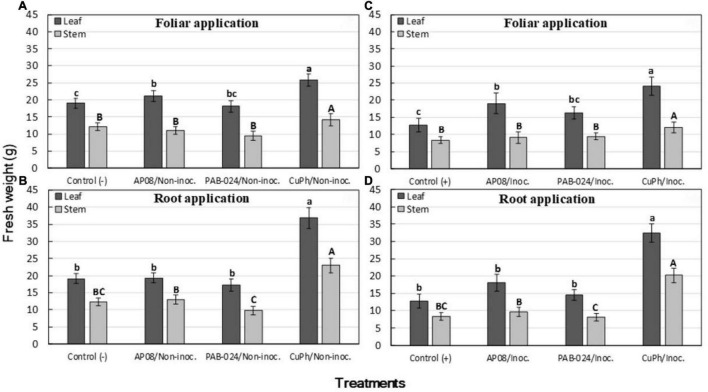
Effect on plant biomass [fresh weight (g) of leaves and stem] in olive plants of cv. Picual, noninoculated **(A,B)** or inoculated **(C,D)** with *Verticillium dahliae*, 3 months after treatment by spray (foliar application) or irrigation (root application) with the following products: water [Control: (–) = noninoculated control; (+) = inoculated control), *Aureobasidium pullulans* (AP08), *Bacillus amyloliquefaciens* (PAB-024) or Copper Phosphite (CuPh). In each graph, columns represent the mean of 30 plants (two experiments with three replicated blocks and five plants per block). Columns with a common uppercase or lowercase letter do not differ significantly according to Fisher’s protected LSD test at *P* = 0.05 for fresh weight of stem and leaf tissues, respectively. Vertical bars are the standard error of the means.

In general, the noninoculated plants showed higher plant biomass than the inoculated plants for all treatments and for the two types of application, as demonstrated by the lack of interaction between the variable inoculation and the other two variables evaluated. The global mean reduction in weight due to inoculation was 16.4%, with 14.5% corresponding to leaves and 18.8% to stems. Likewise, root applications showed a higher general effect on plant biomass than foliar applications for both stem and leaf tissues, but in this case the effect of the treatments depended on the type of application. Since the general ANOVA showed that interactions between the inoculation and the treatments or the type of application were not significant, but the interaction between treatments and type of application was significant, the data on the weight of the plant tissues are presented separately, but the inoculation data for analysis was averaged. The main differences in the effect of the treatments on the plant biomass were observed for the plants treated with CuPh, which showed the highest leaf and stem fresh weight compared with the rest of the treatments for both types of application. The two microorganisms did not show any effect on the plant biomass, except for the foliar treatment with *A. pullulans* AP08, which significantly increased the fresh weight of the leaves compared to the control (Figure 1).

### Effect of Treatments Enhancing the Biochemical Parameters Involved in Plant Resistance

#### Determination and Quantification of Hydrogen Peroxide

**FIGURE 2:**
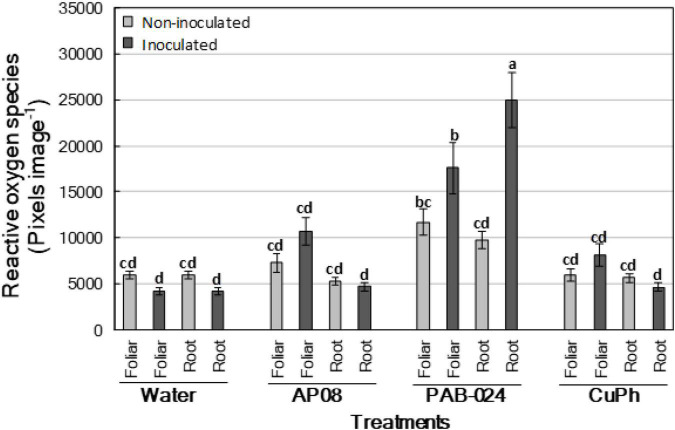
Hydrogen peroxide (H_2_O_2_) staining [reactive oxygen species (ROS); pixels image^– 1^] estimated by using DAB staining in the leaves of olive plants of cv. Picual, which were noninoculated (light gray columns) or inoculated (dark gray columns) with *Verticillium dahliae*, 3 months after treatment by spray (foliar application) or irrigation (root application) with the following products: water (Control), *Aureobasidium pullulans* (AP08), *Bacillus amyloliquefaciens* (PAB-024) and Copper Phosphite (CuPh). Columns represent the mean of 30 plants (two experiments with three replicated blocks and five plants per block each). Columns with common letters do not differ significantly according to Tukey’s HSD test at *P* = 0.05. Vertical bars are the standard error of the means.

The ROS accumulation (H_2_O_2_ staining) in leaves of olive plants of cv. Picual treated with *A. pullulans* AP08, *B. amyloliquefaciens* PAB-024, and CuPh is shown in Figure 2. In general, it can be observed that the ROS is significantly enhanced in the plants treated with *B. amyloliquefaciens* PAB-024, whereas the plants treated with *A. pullulans* AP08 and CuPh did not show significant ROS accumulation in comparison with the control (4,163 ± 438 and 5,920 ± 417 pixels image^–1^ for inoculated and noninoculated treated control plants, respectively).

Results relative to the plants treated with *B. amyloliquefaciens* PAB-024 showed a significant strong accumulation of ROS both in foliar and root applications, as well as in inoculated (17,581 ± 2,748 and 24,972 ± 3,039 pixels image^–1^ for foliar and root applications, respectively) and noninoculated plants (11,690 ± 1,443 and 9,740 ± 916 pixels image^–1^ for foliar and root applications, respectively) compared to the control, with ROS levels found to be always higher in inoculated plants. In the plants treated with *A. pullulans* AP08, only the foliar application notably increased the accumulation of ROS, but in this case it was higher in inoculated plants (10,684 ± 1,450 pixels image^–1^) than in noninoculated ones (7,280 ± 998 pixels image^–1^). Finally, plants treated with PhCu did not show an increase of ROS for any combination of treatments, showing ROS values similar to those observed for the control plants (ROS in inoculated plants = 8,070 ± 1,235 and 4,615 ± 456 pixels image^–1^ for foliar and root applications, respectively; ROS in noninoculated plants = 5,931 ± 693 and 5,617 ± 496 pixels image^–1^ for foliar and root applications, respectively).

The ROS accumulation (H_2_O_2_ staining) was also estimated in roots of olive plants of cv. Picual treated with *A. pullulans* AP08, *B. amyloliquefaciens* PAB-024, and CuPh, but non-significant differences were observed between compounds and control for any combination of treatments (*data not shown*).

#### Evaluation of Hormones Related to Plant Defense

The levels of hormones SA, JA, OPDA, and JA-Ile in leaf, stem and, root samples from olive plants of cv. Picual treated with *A. pullulans* AP08, *B. amyloliquefaciens* PAB-024, and CuPh are shown in Table 3.

**TABLE 3:** Hormone levels (ng per g of dry weight) related to salicylic acid (SA), jasmonic acid (JA), precursor of JA (OPDA), and JA-Isoleucine (JA-Ile) and relative expression of genes involved in the synthesis of acetone cyanohydrin lyase (*ACL*) and lipoxygenase (*LOX*), and the transcription factors involved in SA signal transduction (*WRKY5*) and factors responsive to JA (basic helix-loop-helix; *bHLH*) in leaf, stem and root samples from olive plants of cv.

	Leaf samples									
	
			Hormone levels (ng/g of dry weight)				Relative gene expression*^b^*			
				
Treatment combination			SA	JA	JA-Ile	OPDA	*LOX*	*ACL*	*WRKY5*	*bHLH*
*Aureobasidium pullulans* AP08	Foliar	Non-inoculated	1714.6 ± 615.8 ab	24.5 ± 1.1 ab	70.6 ± 11.7 ab	5.0 ± 0.6 bc	18.4 ± 1.7 a	0.31 ± 0.12 b	6.3 ± 1.5 a	80.7 ± 6.5 a
		Inoculated	1978.5 ± 615.5 ab	10.7 ± 0.7 b	83.5 ± 45.3 ab	30.1 ± 4.0 a	1.6 ± 0.6 b	0.01 ± 0.0 b	0.3 ± 0.1 b	3.4 ± 0.8 b
	Root	Non-inoculated	3818.2 ± 1092.2 ab	23.2 ± 3.3 ab	45.9 ± 12.5 b	0.0 ± 0.0 c	1.0 ± 0.1 b	0.04 ± 0.04 b	0.02 ± 0.01 b	0.8 ± 0.6 b
		Inoculated	2263.9 ± 414.57 ab	44.7 ± 10.0 a	122.2 ± 11.04 a	11.3 ± 5.8 b	2.1 ± 0.9 b	0.01 ± 0.01 b	0.2 ± 0.2 b	0.8 ± 0.6 b
*Bacillus amyloliquefaciens PAB-024*	Foliar	Non-inoculated	651.8 ± 170.4 b	0.0 ± 0.0 c	0.0 ± 0.0 d	0.0 ± 0.0 d	5.0 ± 1.2 b	0.1 ± 0.1 b	0.0 ± 0.0 b	0.7 ± 0.4 b
		Inoculated	3111.3 ± 672.1 ab	0.0 ± 0.0 c	0.0 ± 0.0 d	0.0 ± 0.0 d	2.5 ± 0.6 b	0.02 ± 0.0 b	0.2 ± 0.1 b	0.2 ± 0.1 b
	Root	Non-inoculated	526.8 ± 37.8 b	0.0 ± 0.0 c	0.0 ± 0.0 d	0.0 ± 0.0 d	4.0 ± 1.2 b	0.1 ± 0.1 b	0.02 ± 0.01 b	0.4 ± 0.3 b
		Inoculated	2323.0 ± 656.0 ab	0.0 ± 0.0 c	0.0 ± 0.0 d	0.0 ± 0.0 d	1.1 ± 0.5 b	0.3 ± 0.2 b	0.2 ± 0.04 b	0.3 ± 0.2 b
Copper phosphite (CuPh)	Foliar	Non-inoculated	2519.6 ± 323.8 a	0.0 ± 0.0 c	0.0 ± 0.0 d	0.0 ± 0.0 d	3.3 ± 2.2 b	1.7 ± 0.3 a	1.6 ± 1.1 b	1.1 ± 0.3 b
		Inoculated	2104.8 ± 112.8 ab	0.0 ± 0.0 c	9.1 ± 1.3 c	0.0 ± 0.0 d	0.9 ± 0.4 b	0.02 ± 0.01 b	0.4 ± 0.2 b	0.2 ± 0.1 b
	Root	Non-inoculated	3484.4 ± 748.3 a	0.0 ± 0.0 c	0.0 ± 0.0 d	9.3 ± 2.5 b	0.2 ± 0.1 b	0.1 ± 0.01 b	0.1 ± 0.02 b	0.1 ± 0.1 b
		Inoculated	2275.6 ± 577.1 ab	0.0 ± 0.0 c	0.0 ± 0.0 d	0.0 ± 0.0 d	2.1 ± 1.3 b	0.1 ± 0.1 b	1.2 ± 0.5 b	1.0 ± 0.2 b
Water (control)	Foliar	Non-inoculated	1217.4 ± 430.4 ab	0.0 ± 0.0 c	0.0 ± 0.0 d	0.0 ± 0.0 c	1.8 ± 0.6 b	0.41 ± 0.2 b	0.4 ± 0.3 b	0.4 ± 0.1 b
		Inoculated	2667.5 ± 1310.6 ab	0.0 ± 0.0 c	0.0 ± 0.0 d	1.1 ± 0.3 c	5.5 ± 0.9 b	0.03 ± 0.02 b	2.6 ± 1.6 b	0.2 ± 0.0 b
	Root	Non-inoculated	1217.4 ± 430.4 ab	0.0 ± 0.0 c	0.0 ± 0.0 d	0.0 ± 0.0 d	1.8 ± 0.6 b	0.41 ± 0.2 b	0.4 ± 0.3 b	0.4 ± 0.1 b
		Inoculated	2667.5 ± 1310.6 ab	0.0 ± 0.0 c	0.0 ± 0.0 d	1.1 ± 0.3 c	5.5 ± 0.9 b	0.03 ± 0.02 b	2.6 ± 1.6 b	0.2 ± 0.0 b

			**Stem samples**							
	
			**Hormone levels (ng/g of dry weight)**				**Relative gene expression**			
				
**Treatment combination**			**SA**	**JA**	**JA-Ile**	**OPDA**	***LOX***	***ACL***	***WRKY5***	***bHLH***

*Aureobasidium pullulans* AP08	Foliar	Non-inoculated	103.2 ± 18.5 b	26.6 ± 18.3 a	43.7 ± 18.9 b	18.6 ± 3.4 b	0.3 ± 0.02 a	0.0 ± 0.0 a	0.2 ± 0.1 a	0.03 ± 0.0 a
		Inoculated	99.8 ± 7.6 b	6.2 ± 3.1 a	53.1 ± 10.7 ab	11.0 ± 1.8 b	0.7 ± 0.3 a	0.02 ± 0.01 a	2.3 ± 0.8 a	1.5 ± 0.3 a
	Root	Non-inoculated	84.0 ± 4.4 b	19.7 ± 9.6 a	23.9 ± 8.5 b	21.0 ± 1.3 b	0.5 ± 0.1 a	0.0 ± 0.0 a	0.3 ± 0.2 a	0.2 ± 0.1 a
		Inoculated	86.0 ± 21.2 b	20.9 ± 2.6 a	97.0 ± 17.3 a	8.8 ± 2.8 b	0.8 ± 0.1 a	0.0 ± 0.0 a	0.5 ± 0.2 a	0.2 ± 0.1 a
*Bacillus amyloliquefaciens PAB-024*	Foliar	Non-inoculated	81.2 ± 7.2 b	3.9 ± 2.1 ab	2.4 ± 0.6 c	10.6 ± 1.5 b	0.6 ± 0.3 a	0.4 ± 0.3 a	0.9 ± 0.4 a	12.6 ± 2.7 a
		Inoculated	94.6 ± 5.7 b	0.2 ± 0.03 b	0.2 ± 0.1 cd	11.3 ± 2.2 b	1.3 ± 0.7 a	0.04 ± 0.03 a	0.9 ± 0.2 a	0.2 ± 0.1 a
	Root	Non-inoculated	75.3 ± 8.6 b	0.0 ± 0.0 c	0.0 ± 0.0 d	6.5 ± 1.8 b	0.8 ± 0.2 a	0.02 ± 0.01 a	0.1 ± 0.01 a	0.0 ± 0.0 a
		Inoculated	97.8 ± 12.9 b	5.6 ± 0.8 ab	3.7 ± 0.3 c	17.3 ± 1.2 b	1.8 ± 0.1 a	0.1 ± 0.0 a	2.7 ± 0.3 a	0.1 ± 0.03 a
Copper phosphite (CuPh)	Foliar	Non-inoculated	65.8 ± 6.0 b	0.0 ± 0.0 c	0.4 ± 0.1 c	12.6 ± 4.0 b	0.4 ± 0.3 a	0.1 ± 0.1 a	0.1 ± 0.1 a	17.9 ± 4.0 a
		Inoculated	115.7 ± 13.3 ab	3.0 ± 0.9 b	2.1 ± 0.9 c	79.6 ± 14.2 a	1.2 ± 0.5 a	0.04 ± 0.02 a	1.1 ± 0.4 a	0.1 ± 0.1 a
	Root	Non-inoculated	95.4 ± 11.6 b	0.0 ± 0.0 c	0.0 ± 0.0 d	15.8 ± 2.3 b	1.0 ± 0.1 a	0.2 ± 0.2 a	0.7 ± 0.6 a	0.7 ± 0.4 a
		Inoculated	124.6 ± 18.1 ab	0.2 ± 0.1 b	1.9 ± 0.2 c	7.8 ± 0.2 b	1.0 ± 0.4 a	0.1 ± 0.03 a	1.7 ± 0.8 a	0.1 ± 0.04 a
Water (control)	Foliar	Non-inoculated	85.2 ± 25.1 b	2.8 ± 0.9 b	2.3 ± 0.4 c	18.7 ± 3.1 b	1.4 ± 0.3 a	0.1 ± 0.02 a	1.4 ± 0.6 a	0.1 ± 0.1 a
		Inoculated	178.4 ± 32.6 a	4.8 ± 1.4 ab	6.2 ± 2.1 c	12.5 ± 3.9 b	1.9 ± 0.1 a	0.3 ± 0.3 a	0.5 ± 0.2 a	0.03 ± 0.0 a
	Root	Non-inoculated	85.2 ± 25.1 b	2.8 ± 0.9 b	2.3 ± 0.4 c	18.7 ± 3.1 b	1.4 ± 0.3 a	0.1 ± 0.02 a	1.4 ± 0.6 a	0.1 ± 0.1 a
		Inoculated	178.4 ± 32.6 a	4.8 ± 1.4 b	6.2 ± 2.1 c	12.5 ± 3.9 b	1.9 ± 0.1 a	0.3 ± 0.3 a	0.5 ± 0.2 a	0.03 ± 0.0 a

			**Root samples**							
	
			**Hormone levels (ng/g of dry weight)**				**Relative gene expression**			
				
**Treatment combination**			**SA**	**JA**	**JA-Ile**	**OPDA**	***LOX***	***ACL***	***WRKY5***	***bHLH***

*Aureobasidium pullulans* AP08	Foliar	Non-inoculated	29.4 ± 7.8 ef	7.7 ± 3.5 bc	17.1 ± 2.2 a	35.6 ± 6.6 b	0.1 ± 0.1 c	0.3 ± 0.1 a	15.4 ± 6.5 e	14.7 ± 6.9 b
		Inoculated	48.2 ± 9.2 def	26.2 ± 1.3 a	47.6 ± 5.9 a	75.1 ± 19.0 a	1.3 ± 1.0 c	0.4 ± 0.4 a	320.0 ± 54.5 c	3.7 ± 1.2 b
	Irrigation	Non-inoculated	29.6 ± 7.4 ef	11.8 ± 0.7 bc	28.2 ± 5.5 a	40.4 ± 11.6 b	0.3 ± 0.3 c	0.1 ± 0.03 a	9.0 ± 3.4 e	0.7 ± 0.4 b
		Inoculated	25.9 ± 1.8 ef	21.3 ± 6.3 a	29.8 ± 5.5 a	67.7 ± 16.12 a	2.3 ± 2.0 c	0.1 ± 0.04 a	93.4 ± 30.3 cde	6.3 ± 2.5 b
*Bacillus amyloliquefaciens PAB-024*	Foliar	Non-inoculated	23.6 ± 2.1 ef	6.5 ± 0.3 c	11.7 ± 2.1 a	32.2 ± 6.3 b	368.5 ± 31.0 a	0.7 ± 0.1 a	1951.4 ± 171.5 a	0.04 ± 0.02 b
		Inoculated	31.0 ± 9.2 ef	34.4 ± 10.3 ab	25.3 ± 5.5 a	81.6 ± 13.9 a	47.0 ± 14.2 bc	0.6 ± 0.5 a	293.8 ± 86.7 c	0.3 ± 0.2 b
	Root	Non-inoculated	17.4 ± 2.3 f	3.3 ± 0.6 c	10.1 ± 2.6 a	33.6 ± 13.7 b	8.2 ± 0.6 bc	0.1 ± 0.1 a	154.3 ± 42.9 cde	0.1 ± 0.1 b
		Inoculated	78.1 ± 38.4 cde	12.2 ± 3.1 ab	25.6 ± 10.8 a	37.6 ± 3.7 a	134.8 ± 28.4 ab	1.1 ± 0.5 a	1624.0 ± 228.7 b	5.2 ± 1.1 b
Copper phosphite (CuPh)	Foliar	Non-inoculated	128.7 ± 13.2 abcd	13.4 ± 5.6 c	16.1 ± 5.5 a	43.2 ± 16.6 b	0.5 ± 0.5 c	0.6 ± 0.2 a	62.5 ± 27.7 de	3.2 ± 2.5 b
		Inoculated	162.8 ± 8.8 abc	14.3 ± 3.7 abc	27.1 ± 9.9 a	40.1 ± 19.8 ab	0.01 ± 0.0 c	0.1 ± 0.1 a	65.3 ± 20.1 de	0.5 ± 0.3 b
	Root	Non-inoculated	123.2 ± 14.5 abcd	3.6 ± 2.7 c	7.0 ± 2.6 a	27.5 ± 6.8 b	0.3 ± 0.03 c	0.6 ± 0.2 a	2.4 ± 1.2 e	36.5 ± 21.7 a
		Inoculated	116.7 ± 8.6 bcd	13.3 ± 6.7 abc	20.1 ± 6.6 a	43.1 ± 5.9 ab	0.01 ± 0.0 c	1.1 ± 0.6 a	55.3 ± 13.7 de	0.5 ± 0.3 b
Water (control)	Foliar	Non-inoculated	267.9 ± 46.0 a	2.0 ± 0.8 c	15.7 ± 2.1 a	23.5 ± 4.5 b	0.2 ± 0.03 c	0.1 ± 0.3 a	119.2 ± 44.4 cde	1.3 ± 0.3 b
		Inoculated	318.6 ± 61.8 a	4.4 ± 2.2 c	13.3 ± 1.6 a	50.3 ± 12.8 a	0.01 ± 0.0 c	0.1 ± 0.0 a	261.9 ± 46.5 cd	0.1 ± 0.1 b
	Root	Non-inoculated	267.9 ± 46.0 a	2.0 ± 0.8 c	15.7 ± 2.1 a	23.5 ± 4.5 b	0.2 ± 0.03 c	0.1 ± 0.3 a	119.2 ± 44.4 cde	1.3 ± 0.3 b
		Inoculated	318.6 ± 61.8 a	4.4 ± 2.2 c	13.3 ± 1.6 a	50.3 ± 12.8 a	0.01 ± 0.0 c	0.1 ± 0.0 a	261.9 ± 46.5 cd	0.1 ± 0.1 b

*Picual treated with Aureobasidium pullulans AP08 B. Amyloliquefaciens PAB-024 and copper phosphite (CuPh) by foliar or root applications, and inoculated or noninoculated with Verticillium dahliae strain V-180.*

*^a^For each treatment combination and plant tissue, the data represent the mean of three replicated plants (biological repetitions) and three measurements per simples plant (technical repetitions) ± the standard error of the means. Different letters in the same column represent statistically significant differences according to Tuhey’s HSD test at P = 0.05.*

*^b^Relative gene expression in comparison with the Olive act-1 gene (Schilirò et al., 2012).*

Treatments with *A. pullulans* AP08 were able to stimulate the levels of SA mainly in the leaves, with the higher values being observed in the noninoculated plants treated by root application. However, none of the values observed were significantly different from those observed in the positive controls as well as in the rest of the treatments. The treatment with *B. amyloliquefaciens* PAB-024 only showed an enhancement of the SA content mainly in leaves from inoculated plants, whereas those noninoculated showed low levels of SA. Finally, the CuPh increased the levels of SA in all the treatments, regardless of the plant tissue, the type of application or the presence of *V. dahliae*.

The levels of JA were significantly stimulated in comparison to the controls only in certain tissues and treatment combinations with *A. pullulans* AP08 or *B. amyloliquefaciens* PAB-024. Interestingly, the plants treated with *A. pullulans* AP08 showed a significant strong enhancement of JA both in foliar and root applications as well as in inoculated and noninoculated plants. In detail, it can be observed that *A. pullulans* AP08 is able to induce a systemic enhancement of JA, regardless of the plant tissue, but especially in leaves. In the same way, the treatment with *B. amyloliquefaciens* PAB-024 was also able to increase the levels of JA; however, this enhancement is important only in the roots of treated and inoculated plants. The treatment with CuPh increases the levels of JA in inoculated plants treated by spraying, regardless of the plant tissue, but it did not show significant differences compared to the control.

Regarding the metabolites present in the JA pathway, in general, an increase of OPDA in roots of inoculated plants was observed compared with control plants in all cases. This increase can also be observed in the plants treated with *A. pullulans* AP08 both by foliar or root applications, in which the inoculated plants showed significantly higher levels than the noninoculated ones. In general, the treatment with *B. amyloliquefaciens* PAB-024 showed similar levels of OPDA to those observed in positive control plants. However, inoculated plants treated by spraying showed significantly higher levels than the rest of the bacteria treated plants. Minor or non-significant differences were observed in the levels of OPDA in plants treated with CuPh compared with the positive controls.

Finally, the treatment with *A. pullulans* AP08 strongly increases the presence of JA-ile, which is the bioactive metabolite from JA pathway, in all the plant tissues evaluated. According to the results observed with the JA, the treatment with the *B. amyloliquefaciens* PAB-024 was also able to induce the JA-ile, but this enhancement is only present in the stem and roots of both inoculated and inoculated plants, but without significant differences with the controls. In the same way, minor or non-significant differences were observed in the levels of JA-ile in plants treated with CuPh in comparison with the positive controls.

#### Validation and Expression Profile Analysis by Quantitative Real-Time PCR

The relative expression of *ACL*, *LOX*, *WRKY5*, and *bHLH* genes in leaf, stem, and root samples from olive plants of cv. Picual treated with *A. pullulans* AP08, *B. amyloliquefaciens* PAB-024, and CuPh are shown in Table 3.

The expression of genes related to the SA (*ACL* and *WRKY5*) showed a discrete enhancement in all the treatments. Exceptionally, a remarkable enhancement of the transcription factor *WRKY5* was observed in the roots of the inoculated plants treated by root application, and on the noninoculated plants treated by foliar application with *B. amyloliquefaciens* PAB-024. On the other hand, *ACL* expression shows similar levels to those observed in negative controls for all treatment combinations and tissues.

Regarding the gene expression related to the JA (*LOX* and *bHLH*), it can be observed that the *LOX* gene is strongly enhanced in the plants treated with *B. amyloliquefaciens* PAB-024, whereas in the rest of the treatments the expression of this gene at this timepoint is low. However, the expression of the transcription factor *bHLH* is enhanced in the different treatments. Results showed that in plants treated with *A. pullulans* AP08 the treatment enhances high levels of *LOX* and *bHLH* expression in the leaves from the noninoculated and foliar treated plant, as well as high levels of *bHLH* expression in the roots from noninoculated and foliar treated plants. On the other hand, plants treated with *B. amyloliquefaciens* PAB-024 showed an increase of *bHLH* levels only in the stem of foliar treated and noninoculated plants, and in the roots of inoculated plants treated by root applications. Finally, plants treated with PhCu showed an increase of this gene mainly in noninoculated plants.

## Discussion

This study was conceived to shed light on the biochemical and genetic responses associated with HRI that could take place during the interaction among biocontrol treatments, a woody host (olive), and a vascular pathogen (*V. dahliae*). In this work, two microorganisms (*A. pullulans* AP08 and *B. amyloliquefaciens* PAB-024) and a phosphonate salt copper phosphite (CuPh) were selected for their high effectiveness previously observed against *V. dahliae* in olive plants treated by foliar or root applications before pathogen infection (López-Moral et al., 2021). From this preliminary study, we had the hypothesis that these compounds could be acting by inducing the resistance of the plant rather than a direct effect on the pathogen. Since the induction of defense is usually a systemic response of the plant, is not necessary to apply the treatment in the same tissue as the pathogen. With this rationale, it is interesting to ascertain if the foliar treatments are effective in extensive crops like olive in which the soil treatments are much more difficult due to the lack of an irrigation system. Thus, the present study is useful to clarify the mechanism of action of these compounds and to determine their role in enhancing the olive immune system against infections by *V. dahliae*.

The effect of the different compounds in the disease reduction was in concordance with those previously described by López-Moral et al. (2021). Thus, in general, root applications resulted in higher effectiveness than foliar applications. Considering the type of application, *A. pullulans* AP08 was the most effective compound when it was applied by foliar applications, whereas CuPh was the most effective by root application. In addition, treatments with CuPh resulted in a significantly higher fresh weight of the plants compared with the rest of the treatments, which suggests their potential effect by biostimulation as previously supported by Gómez-Merino and Trejo-Téllez (2015). Subsequently, the hormonal pathways and the related gene expression were analyzed to ascertain the implication of the defensive pathways in the enhancement of the HRI by the evaluated compounds. Interestingly, different results were obtained, depending on the compound, type of application or inoculation treatment combinations.

It is well known that plant defense against pathogen infection is regulated by the hormones SA, JA, and ethylene (Llorens et al., 2017b; Montes-Osuna and Mercado-Blanco, 2020). The SA pathway is responsible to control the defense against biotrophic pathogens; however, its activation is also required for effective plant defense against hemibiotrophic pathogens such as *V. dahliae.* In this work, high levels of SA were detected on leaves from all treatment combinations, but without significant enhancements compared to the nontreated controls. Considering the gene expression linked to salicylic acid, only the relative *WRKY5* gene expression showed a strong enhancement in olive plants treated with *B. amyloliquefaciens* PAB-024. Our results are not at all in concordance with those shown by Gharbi et al. (2016), who linked the early activation of SA with an effective resistance to *V. dahliae* reducing wilt symptoms. These authors monitored the expression of SA related genes *PR10, PR1, PR5, PLD*, and *WRKY5* at early time points (from 1 to 30 days) showing that the higher expression of this pathway is around 8–15 days after infection, and a progressive reduction since then (Gharbi et al., 2016). These results are interesting since it has been previously described that the constitutive overexpression of *GbMPK3* (Long et al., 2020) or *GhWRKY70* (Xiong et al., 2019) enhance the SA-mediated defense pathway genes which produced an increased susceptibility to *V. dahliae*, probably due to the negative crosstalk between the JA/SA pathways. Under our conditions, the implication of SA is not so evident since the evaluation of hormones and gene expression was performed 3 months after inoculation, which could skip the biotrophic phase of the fungi. Nevertheless, it is worth mentioning that plants treated with *B. amyloliquefaciens* PAB-024 showed high levels of ROS accumulation, which simultaneously accompany the up-regulation of SA-related genes (Llorens et al., 2017a). It has been widely studied that against pathogens infection, there is an increase in SA levels preceded by apoplastic H_2_O_2_ bursts. This mechanism suggests that H_2_O_2_ originated in chloroplasts and peroxisomes triggers SA biosynthesis, which is essential for main defense responses: transcriptional reprogramming, cell death, and stomatal closure (Herrera-Vásquez et al., 2015)

Regarding the JA, which is the hormone related to the defense against necrotrophic pathogens, our results show a strong accumulation of Ja and Ja-ile in plants treated with *A. pullulans* AP08 in all tissues analyzed, as well as in plants treated with *B. amyloliquefaciens* PAB-024 and CuPh in roots. These findings could suggest an implication of the JA in the induced resistance by the tested compounds. Our results are in concordance with those previously shown by Gharbi et al. (2017), who reported that the JA, as well as the expression of the genes responsive to this hormone, could be related to the higher resistance of olive trees to *V. dahliae*. In addition, these authors also observed that the activation of JA pathway is produced later than the SA response, probably when the necrotrophic phase of the pathogen starts. Our results agree with this hypothesis since at 3 months after inoculation there is a strong accumulation of JA and JA-related metabolites. The relation of JA with the resistance against *V. dahliae* has been thoroughly reported in several plant species. Xiong et al. (2020) demonstrated that the downregulation of *GhWRKY70D13* in cotton plants increased the accumulation of JA and JA-ile which enhanced resistance to *V. dahliae*. In the same way, Luo et al. (2021) observed that phosphate deficiency in cotton induced the activation of JA biosynthesis and accumulates a series of anti-fungal flavonoids which, in turn, confer protection against this fungus. Moreover, it is interesting to note that the olive resistance against *V. dahliae* mediated by the JA pathway could be also linked with the cultivar resistance. Likewise, studies conducted by Gharbi et al. (2017) demonstrated the expressión of *JZIM* (jasmonate ZIM domain) and *bHLH* genes were strongly induced in olive of cv Sayali but not in “Chemlali” (susceptible) (Gharbi et al., 2017). Altogether suggest that the JA pathway could play an important role in inducing the plant defenses in olive against *V. dahliae*, which could be modulated by the genotype, the application of certain biocontrol treatments, and probably, also by the combined effect of both genotype and biocontrol treatments.

In addition, accumulation of callose was also evaluated in this study, following the protocol described by Llorens et al. (2017a), but no levels of callose deposition could be observed for any treatment and control (*data not shown*). This fact makes sense in our experimental conditions for two main reasons: (i) we evaluated all the parameters related to HRI at 3 months after inoculation, but callose accumulation is a cellular response of early post-invasive defenses based on the generation of physical barriers in the side of infection to prevent the pathogen colonization and typically triggered by conserved pathogen-associated molecular patterns (PAMPs) (Luna et al., 2011; Voigt, 2014; Llorens et al., 2017a); and (ii) the biology of *V. dahliae*, that infects olive through the roots by microsclerotia germination mediated by root exudation, did not match with the biotrophic pathogens, i.e., powdery mildew that usually enhances the plant responses to induce callose deposition (Ellinger et al., 2013; Llorens et al., 2017a)

Summarizing, several transcription factors were previously shown to play-role in plant defense in olive against *V. dahliae* (Schilirò et al., 2012) such as *LOX*, *ACL*, *bHLH*, and *WRKY5* have been shown in this present study to be induced in olive plants after different biological treatments with the presence or absence of the pathogen. Up-to-date, their role in the modulation of HRI in olive is still uncertain. Therefore, this study helps us to reinforce the hypothesis that the three compounds tested here can act as HRI in olive against *V. dahliae*. This fact can be concluded mainly in the case of *A. pullulans* AP08, which resulted in low effectiveness inhibiting mycelial growth and MS germination of *V. dahliae in vitro*, but in higher effectiveness reducing the disease progress in previous studies conducted by López-Moral et al. (2021), showing also high levels of JA and metabolites and genes present in the JA pathway in this study. Thus, these findings could suggest an implication of the JA in the HRI by some of the tested compounds. The knowledge generated in this work results in an important step toward selecting potential candidates for effective biocontrol of VWO. To continue this study, new insights into the effect of these three compounds as HRI over time should be conducted to determine the durability of their effect inarching the olive defenses against *V. dahliae*.

## Conclusion

In general, root applications resulted in higher effectiveness in reducing the disease progress than foliar applications. *A. pullulans* AP08 was the most effective compound when it was applied by foliar applications, whereas CuPh was the most effective by root application. Treatments with CuPh resulted in a significantly higher fresh weight of the inoculated and noninoculated plants compared with the rest of the treatments, suggesting their potential fertilizer effect *in planta*. Concerning the effect of compounds enhancing HRI in olive, high levels of SA were detected on leaves from all treatment combinations, but without significant enhancements compared to the nontreated control. ROS was significantly enhanced in plants treated with *B. amyloliquefaciens* PAB-024 compared to the remaining treatments and control. Considering the gene expression linked to salicylic acid, only the relative *WRKY5* gene expression showed a strong enhancement in olive plants treated with *B. amyloliquefaciens* PAB-024. Interestingly, a strong accumulation of JA and JA-ile was observed in plants treated with *A. pullulans* AP08 in all the tissues analyzed as well as in plants treated with *B. amyloliquefaciens* PAB-024 and CuPh in roots. These findings could suggest an implication of the JA in the HRI by the tested compounds. Finally, callose accumulation was not observed in any tissue for any treatment and control, probably for the experimental conditions as well as for the pathosystems used in this study. The knowledge generated in this work results in an important step toward selecting potential candidates for an effective biocontrol of VWO.

## Data Availability Statement

The original contributions presented in the study are included in the article/supplementary material, further inquiries can be directed to the corresponding author.

## Author Contributions

AL-M, AT, CA-B, and EL conceived and designed the study. AL-M and CA-B performed the *in planta* experiments and wrote the original draft of the manuscript. AL-M, EL, and LS analyzed the parameters involved in induction resistance. AL-M, CA-B, and EL analyzed the data. AT, EL, and PG-A reviewed the manuscript. AT and PG-A acquired funding support. All authors contributed to the manuscript and approved the submitted version.

## Conflict of Interest

The authors declare that the research was conducted in the absence of any commercial or financial relationships that could be construed as a potential conflict of interest.

## Publisher’s Note

All claims expressed in this article are solely those of the authors and do not necessarily represent those of their affiliated organizations, or those of the publisher, the editors and the reviewers. Any product that may be evaluated in this article, or claim that may be made by its manufacturer, is not guaranteed or endorsed by the publisher.

## Footnotes

1
http://www.scharlab.com

2
http://www.micromass.co.uk

3
http://www.omegabiotek.com

4
http://www.promega.com
